# Delayed crystallization of ultrathin Gd_2_O_3 _layers on Si(111) observed by *in situ *X-ray diffraction

**DOI:** 10.1186/1556-276X-7-203

**Published:** 2012-03-29

**Authors:** Michael Hanke, Vladimir M Kaganer, Oliver Bierwagen, Michael Niehle, Achim Trampert

**Affiliations:** 1 Paul-Drude-Institut für Festkörperelektronik, Hausvogteiplatz 5-7, Berlin, D-10117, Germany

## Abstract

We studied the early stages of Gd_2_O_3 _epitaxy on Si(111) in real time by synchrotron-based, high-resolution X-ray diffraction and by reflection high-energy electron diffraction. A comparison between model calculations and the measured X-ray scattering, and the change of reflection high-energy electron diffraction patterns both indicate that the growth begins without forming a three-dimensional crystalline film. The cubic bixbyite structure of Gd_2_O_3 _appears only after a few monolayers of deposition.

## Background

Binary rare-earth metal oxides became increasingly important as gate dielectrics in metal oxide semiconductor field-effect transistor technology [1,2] and as catalysts. The application of these oxides as gate dielectrics is based on their high dielectric constants [3], large bandgaps [4], high thermal stability of their interfaces to silicon [5], and large band offsets with silicon. Gd_2_O_3 _is a promising candidate among the lanthanide oxides due to its close lattice match to silicon ($a_{\text{G}{\text{d}}_{2}{\text{O}}_{3}}=10.812Å;$ 2*a*_si _= 10.862 Å). Epitaxial growth of Gd_2_O_3 _has been demonstrated for a variety of substrates, including GaN [1], Si [6], and SiC [7] by atomic layer deposition [8] and molecular beam epitaxy (MBE) [9].

Here, we present an MBE growth study of Gd_2_O_3 _on Si(111), applying synchrotron-based X-ray diffraction with particular focus on the formation of the very first crystalline layers. These measurements were done during sample growth *in situ*, in a continuous way without any growth interruption.

## Methods

### Molecular beam epitaxy

All samples were grown in a dedicated MBE chamber under ultrahigh vacuum (UHV) conditions. The 2 × 2-cm^2 ^Si(111) substrates were prepared by a 10-min dilute (5%) HF treatment, to remove the thermal oxide, followed by a 10-min H_2_O rinse. Next, they were loaded into the MBE system, degased at 300°C in the load lock for 20 min, and annealed in the growth chamber under UHV at 720°C (measured using a thermocouple between heater and substrate) for 60 min to prepare a (7 × 7) reconstructed Si(111) surface. The sample temperature was subsequently ramped to the growth temperature of 700 C. During the growth, Gd_2_O_3 _powder (99.999%) was evaporated from a special effusion cell (TUBO), which allows for cell temperatures in excess of 2,000°C [10]. Temperature and flux from this cell can be adjusted more accurately than with conventional e-beam evaporators. Throughout the experiment, the cell temperature was kept constant at 1,650°C (thermocouple temperature; the actual temperature of the source material is higher), resulting in a growth rate of 0.13 Å/min. Molecular oxygen at 10^-7 ^mbar was added 30 s prior to the growth sequence and throughout the entire layer deposition (OB, unpublished work).

### X-ray diffraction

The X-ray scattering experiments were performed at the dedicated beamline U125/2-KMC at the synchrotron BESSY II (Helmholtz-Zentrum Berlin HZB, Berlin, Germany). This experimental setup [11] combines the MBE system described above with the ability to perform high-resolution X-ray diffraction, which enables unique *in situ *growth studies. The primary X-ray beam with a size of 500 × 500 μm^2 ^enters and exits the growth chamber via X-ray transparent Be windows, whereas the scattered intensity can be probed in an angular range of 0° to 120° in-plane and 0° to 50° out-of-plane. Both the sample and the detector can be precisely moved during the growth to probe scattered intensity in a wide range of reciprocal space. An X-ray energy *E *of 12 keV, selected by a Si(111) double crystal monochromator (Δ*E */*E *= 10^-4^), is chosen as a compromise between the accessible area in reciprocal space and primary intensity. Throughout the experiment, the incidence angle was kept constant at *α_i _*= 0.2°.

## Results and discussion

We index the reflections in surface (hexagonal) coordinates, marked with (*HKL*)_hex_, instead of using bulk indices, denoted by (*HKL*)_cub _[12]. Referring to the symmetry of the Si(111)_cub _substrates, we define the surface unit cell by vectors **a**, **b**, and **c **with lengths *a *= *b *= 3.84 Å, *c *= 9.405 Å and *α *= *β *= 90°, *γ *= 120°. For example, the Si(022)_cub _bulk reflection refers to (104)_hex _in surface coordinates.

### Post-growth X-ray diffraction

**Figure 1:**
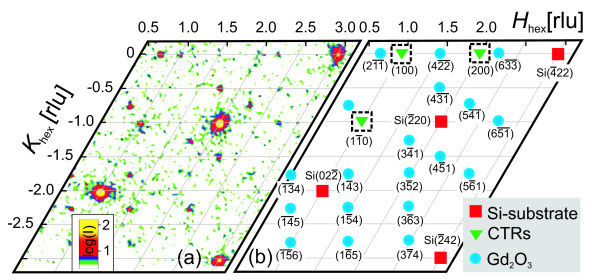
**In-plane intensity distribution in reciprocal space**. (**a**) Measured and (**b**) schematic in-plane intensity distribution showing the Gd_2_O_3 _bixbyite (blue circles) and substrate (red squares) reflections, and the tails of the CTRs (10*L*)_hex_, (20*L*)_hex_, and ${\left(1\bar{1}L\right)}_{\text{hex}}$ (green triangles).

Figure 1a shows a post-growth in-plane intensity distribution of a 13-monolayer (ML, 1 ML = 3.12 Å)-thick Gd_2_O_3 _layer on Si(111) measured by *in situ *X-ray diffraction. All reflections are labeled in the sketch (Figure 1b) which also addresses their different origin. There are basically three types of reflections: intense substrate peaks (red squares), layer reflections (blue circles), and intensity tails from crystal truncation rods (CTRs, green triangles), which are forbidden reflections for bulk Si. This diffraction pattern corresponds to the cubic bixbyite structure (space group 206) of Gd_2_O_3 _with single orientation, in agreement with previous studies, which found an A-B twinning relation for Gd_2_O_3_/Si(111) heteroepitaxy [13]. Thus, the layer is 180° rotated with respect to the underlying substrate, referring to the Si-CaF_2_-type interface with the silicon atoms directly bonded to calcium [14].

### *In-situ *X-ray studies during growth

**Figure 2:**
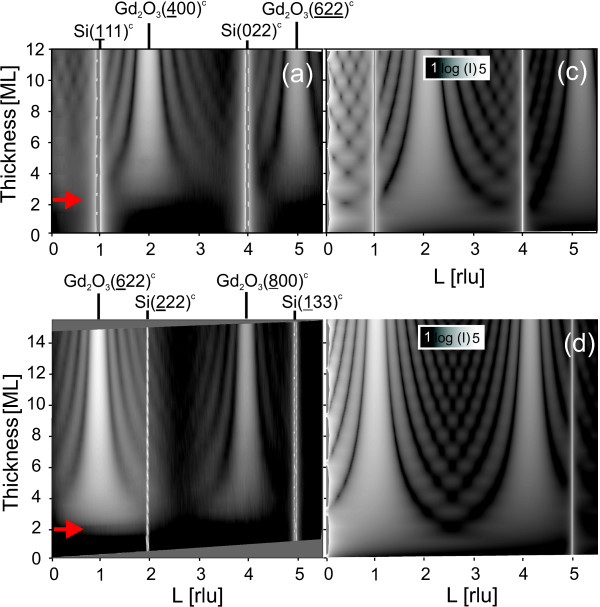
**Out-of-plane intensity distribution in reciprocal space**. Intensities along the CTRs (10*L*)_hex _and (20*L*)_hex _measured *in situ *(**a, b**) and calculated (**c, d**) as a function of nominal layer thickness. Each CTR intersects two substrate reflections. The red arrows point to the beginning of crystal layer formation.

To study the epitaxial growth of Gd_2_O_3 _on Si(111) *in situ*, we probed the evolution of the out-of-plane scattering by measuring three different CTRs, whose positions are indicated by green triangles in Figure 1b. Figure 2 shows measured (a, b) and calculated (c, d) intensity distributions along the CTRs (10*L*)_hex _and (20*L*)_hex _for layer thicknesses of up to 14 ML. The third measured CTR, ${\left(1\bar{1}L\right)}_{\text{hex}}$, is not shown. Since the growth was continuous without any interruption, the thickness changes during an individual CTR scan by approximately 0.6 ML. The depicted scan range in *L *covers two silicon peaks, namely $\text{Si}{\left(1\bar{1}1\right)}_{\text{cub}}$ and Si(022)_cub _at the CTR (10*L*)_hex_, as well as $\text{Si}{\left(\bar{2}22\right)}_{\text{cub}}$ and $\text{Si}{\left(\bar{1}33\right)}_{\text{cub}}$ at the CTR (20*L*)_hex_. Due to the A-B twinning relation to the substrate, the layer peaks do not coincide with those of the substrate. Gd_2_O_3 _in its bixbyite structure exhibits a lattice parameter *a *of 10.812 Å (1 ML in [111] direction refers to $a/2\sqrt{3}=3.12\;Å$), which nearly equals the substrate value. The resulting lattice mismatch is as small as 0.4%. Such a type of layer (closely lattice-matched and A-B-twinned with respect to the substrate) will excite reflections shifted from substrate contributions by *L *= ± 1 as shown in Figure 2. The onset of these layer reflections sensitively probes the appearance of a three-dimensional crystalline Gd_2_O_3 _lattice.

### Scattering calculations

**Figure 3:**
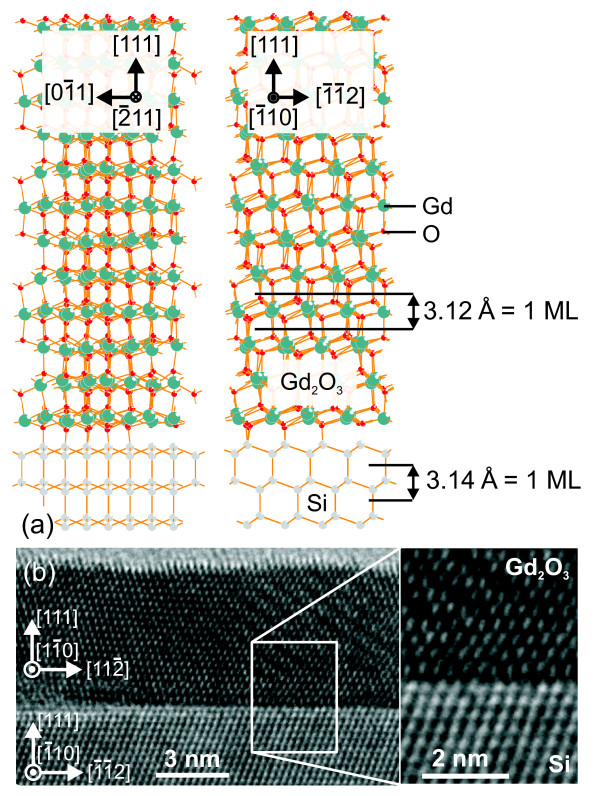
**Real-space structure of Gd_2_O_3 _on Si(111)**. (**a**) Structure model shown in two different azimuths $\left[0\bar{1}1\right]$ and $\left[\bar{1}\bar{1}2\right]$. (**b**) A transmission electron micrograph of the layer and the interface region.

For comparison, the CTR intensities were calculated using the structure model shown in Figure 3a. The semi-infinite silicon substrate and a finite number of the oxide layers, in a twinned orientation of the ABCABC packing with respect to the substrate, were included in the calculation. The intensity calculations were performed in the distorted wave Born approximation [15] since the incidence angle is close to the critical angle. A non-integer deposition was calculated as a sum of scattering amplitudes of two films, with the integer numbers of monolayers smaller and larger than the actual deposition, taken in the appropriate proportion. The time-dependent layer thickness was determined from the current growth time. The growth rate was obtained by dividing the total film thickness, determined from a post-growth CTR measurement, by the total growth time.

### Layer formation at the interface

**Figure 4:**
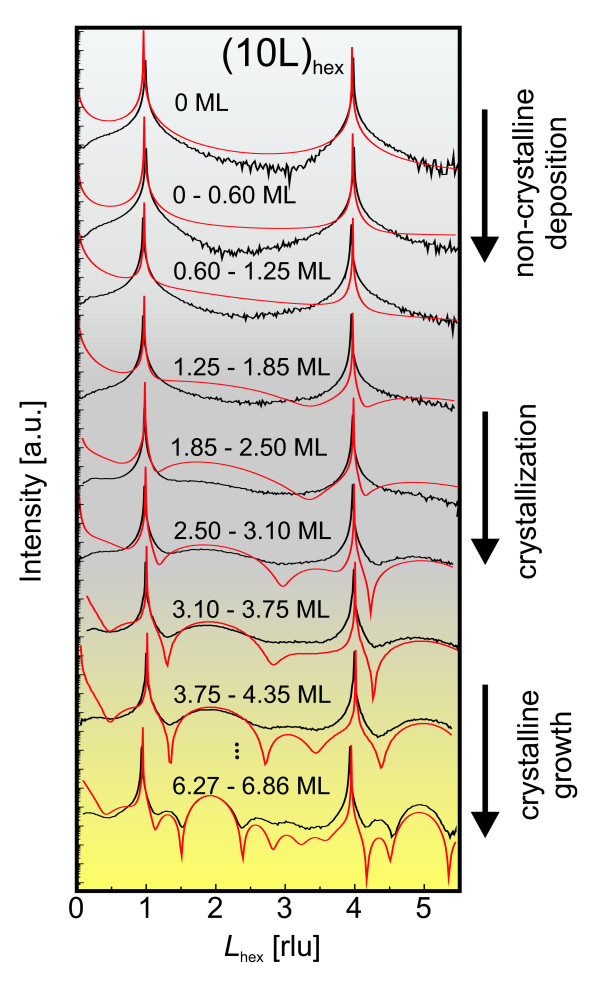
**Comparison between measured and simulated CTRs**. One-dimensional intensity profiles along the CTR (10*L*)_hex _as measured (black curves) and calculated (red). The given values refer to nominally deposited layer thickness. In the beginning, the experimental profile does not change (gray), followed by a crystallization process (dark gray) which finally ends up with crystalline growth (yellow).

The measured (Figure 2a, b) and calculated (Figure 2c, d) intensity distributions agree for a layer thickness larger than about 3 ML (indicated by red arrows in the figure). Layer peaks become stronger and are subsequently accompanied by thickness fringes whose separation in *L *shrinks with increasing thickness. However, in contrast to the calculations, which contain a fingerprint of a three-dimensional crystalline Gd_2_O_3 _lattice already from the first monolayer on, there is no experimental evidence for a crystalline layer below the deposition of 2 to 3 ML. For a more detailed analysis, Figure 4 compares a selection of measured and calculated CTRs at different deposition thicknesses. The measured CTRs do not show an indication of the crystalline film for the deposition less than 2 ML, while the intensity changes due to the film are visible on the calculated CTRs from the very beginning of the growth. We cannot exclude an in-plane ordering in separate layers without any registry among one another. At depositions between 2 and about 4 ML, the measured curves become more and more close to the calculated ones, showing that the crystalline order develops in the film. For the film thickness larger than 4 ML, a crystalline film grows, and the thickness determined from the fringes on the CTRs agrees with the nominally deposited film thickness. This agreement proves that the sticking coefficient is constant for the entire growth starting from the layer-substrate interface - consistent with a constant deposition rate.

Atomic force microscopy of the pure Si(111) substrate (not shown) yields a mean terrace size of 600 nm, which does not change after the deposition of 1 and 2 ML of Gd_2_O_3_. The roughness of 0.46 nm is preserved during growth. Post-growth X-ray reflectivity measurements prove the nominal layer thickness with an RMS roughness of less than 1 ML.

The evolution of the reflection high-energy electron diffraction (RHEED) pattern (not shown) during the first MLs is consistent with the 'delayed crystallization' scenario: During deposition of the first ML, the 2D streaky RHEED pattern from the Si(111) surface disappeared completely and transformed into a diffuse RHEED pattern with almost vanishing specular spot intensity - indicative of the lack of crystal order [13]. The specular spot intensity recovered after the deposition of (3 to 5 ML) together with the onset of pronounced RHEED oscillations.

For the final film, the fringes of CTRs (sensitive to crystalline film thickness only) and the X-ray reflectivity fringes (sensitive to total film thickness irrespective of crystalline order) correspond to the same thickness. Therefore, the crystalline order developed over the whole film thickness, without leaving a noncrystalline initial layer at the interface. This conclusion is in agreement with cross-sectional transmission electron micrographs in Figure 3b which demonstrates a pseudomorphic film and an atomically flat layer-substrate interface.

## Conclusions

We have performed a synchrotron-based *in situ *X-ray diffraction study during the epitaxial growth of Gd_2_O_3 _on Si(111). By comparing measured crystal truncation rods to X-ray scattering calculations, we found that the crystallization into three-dimensional cubic bixbyite structure was delayed: it started at a layer thickness of about 2 ML and proceeded up to 4 ML.

## Abbreviations

CTR: crystal truncation rod; MBE: molecular beam epitaxy; ML: monolayer; RHEED: reflection high-energy electron diffraction; TEM: transmission electron microscopy; UHV: ultrahigh vacuum.

## Competing interests

The authors declare that they have no competing interests.

## Authors' contributions

MH performed the X-ray scattering experiment. VMK carried out the scattering simulations. The samples were grown by OB. MN and AT performed the TEM analysis. All authors read and approved the final manuscript.
